# Super-resolution vibrational microscopy by stimulated Raman excited fluorescence

**DOI:** 10.1038/s41377-021-00518-5

**Published:** 2021-04-20

**Authors:** Hanqing Xiong, Naixin Qian, Yupeng Miao, Zhilun Zhao, Chen Chen, Wei Min

**Affiliations:** Department of Chemistry, Columbia University, New York, NY 10027 USA

**Keywords:** Super-resolution microscopy, Raman spectroscopy, Multiphoton microscopy

## Abstract

Inspired by the revolutionary impact of super-resolution fluorescence microscopy, super-resolution Raman imaging has been long pursued because of its much higher chemical specificity than the fluorescence counterpart. However, vibrational contrasts are intrinsically less sensitive compared with fluorescence, resulting in only mild resolution enhancement beyond the diffraction limit even with strong laser excitation power. As such, it is still a great challenge to achieve biocompatible super-resolution vibrational imaging in the optical far-field. In 2019 Stimulated Raman Excited Fluorescence (SREF) was discovered as an ultrasensitive vibrational spectroscopy that combines the high chemical specificity of Raman scattering and the superb sensitivity of fluorescence detection. Herein we developed a novel super-resolution vibrational imaging method by harnessing SREF as the contrast mechanism. We first identified the undesired role of anti-Stokes fluorescence background in preventing direct adoption of super-resolution fluorescence technique. We then devised a frequency-modulation (FM) strategy to remove the broadband backgrounds and achieved high-contrast SREF imaging. Assisted by newly synthesized SREF dyes, we realized multicolor FM-SREF imaging with nanometer spectral resolution. Finally, by integrating stimulated emission depletion (STED) with background-free FM-SREF, we accomplished high-contrast super-resolution vibrational imaging with STED-FM-SREF whose spatial resolution is only determined by the signal-to-noise ratio. In our proof-of-principle demonstration, more than two times of resolution improvement is achieved in biological systems with moderate laser excitation power, which shall be further refined with optimized instrumentation and imaging probes. With its super resolution, high sensitivity, vibrational contrast, and mild laser excitation power, STED-FM-SREF microscopy is envisioned to aid a wide variety of applications.

## Introduction

The advent of far-field super-resolution fluorescence microscopy^1–4^ has greatly sharpened our vision in the microscopic world over the past two decades^4–6^. Distinct from fluorescence spectroscopy, Raman spectroscopy offers significantly higher chemical specificity of the molecular systems^7,8^. Indeed, the Raman spectrum is, in general, 50–100 times narrower than the fluorescence spectrum in condensed phase at room temperature. Hence, pushing Raman microscopy beyond the optical diffraction limit should greatly enrich the molecular information one can obtain from nanoscale systems.

Despite the perceived importance and extensive research efforts^9–18^, true super-resolution (defined as diffraction-*unlimited*) Raman imaging in the optical far-field remains a major technical challenge especially in biological environments, largely because the conventional Raman scattering is intrinsically much weaker (i.e., insensitive) when compared to fluorescence. Quantitatively, depending on the extent of electronic resonance, the Raman scattering cross-section of a typical chemical bond is 8–14 orders of magnitude smaller than the absorption cross-section of a typical chromophore^19^. As a consequence, super-resolution vibrational imaging methods based on excitation saturating^9,10^, depleting^11–14^, or high-order nonlinearity^16^ of the Raman transitions all require extremely intense laser power in order to achieve a moderate resolution improvement (often less than a factor of 2).

Most recently, we have developed a ultrasensitive vibrational spectroscopy called stimulated Raman excited fluorescence (SREF)^20,21^, which enables all-far-field Raman spectroscopy with sensitivity down to the single-molecule level for the first time. SREF spectroscopy has found exciting applications in biophotonics, biophysics, and materials science where the exquisite vibrational specificity and sensitivity are harnessed for advanced sensing or super-multiplexed imaging^20,22,23^. In the current work, we leveraged the high chemical specificity and detection sensitivity of SREF spectroscopy and exploited its potential for high-contrast super-resolution vibrational imaging. We first showed that the inevitable anti-Stokes fluorescence background of the SREF spectrum hinders the direct application of existing super-resolution strategies on SREF imaging. We then solved this background problem by applying a frequency-modulation (FM) strategy achieved through self-phase modulation in optical fiber^24^. As such, background-free FM-SREF imaging was achieved with high vibrational contrast in biological samples stained with chemical probes. Further employing stimulated emission depletion (STED) on the fluorescence detection step of the SFEF process, we achieved STED-FM-SREF microscopy for super-resolution vibrational imaging. A resolution improvement of more than two times was observed for a variety of biological systems. In addition to instrumentation, new SREF probes suitable for specific labeling were also developed to facilitate the biomedical application of this technique. We envision STED-FM-SREF microscopy to aid a wide variety of applications such as vibrational sensing and super-multiplexed imaging.

## Result

### Difficulty of direct coupling of STED with SREF imaging

**Fig. 1:**
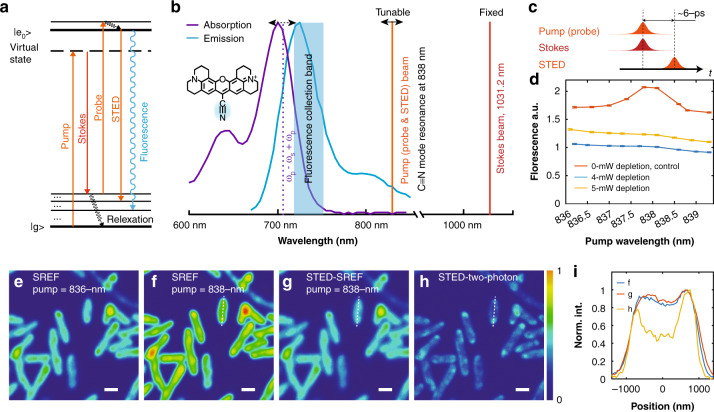
Direct coupling of stimulated emission depletion (STED) and stimulated Raman excited fluorescence (SREF). **a** Energy diagram of STED-SREF. **b** Spectroscopy configuration of STED-SREF. The nitrile mode of Rhodamine 800 (Rh800) is used here. **c** Temporal sequence of the pulses used for STED-SREF. The pump pulse, probe pulse, and STED pulse share the same wavelength. All laser pulse durations are 2 ps, and the pulse repetition rate is 80 MHz. **d** SREF spectra of the Rh800 nitrile mode under different STED beam power. Red curve, blue curve and orange curve are corresponding to 0, 4, and 5 mW, respectively. The STED beam profile is Gaussian here. Error bars represent 95% confidence intervals of the mean values of normal distributions fitted by 200 independent measurements. **e**, **f** Corresponding to SREF imaging of Rh800-stained *E. coli* cells with pump wavelengths set at 836 nm (off resonance of the nitrile mode) and 838 nm (vibrational resonance of the nitrile mode), respectively. **g** STED-SREF imaging with pump wavelengths set at 838 nm. **h** The STED two-photon fluorescence image of the same field of view, with two-photon fluorescence excited by Stokes beam. The Stokes beam is modulated so that the demodulated signal shown in (**h**) represents the pure two-photon fluorescence. **i** The normalized intensity distributions along the corresponding dash lines in (**f**–**h**). For (**d**–**h**), the pump (probe) power was set to 12 mW, Stokes power was set to 10 mW; For (**g**–**h**), the STED beam was transformed to the donut shape and was set to 5 mW. Scale bar: 1 µm

In SREF excitation, a fluorophore is first excited by stimulated Raman excitation to the target vibrational excited state (orange line and red line in Fig. 1a, b), then a probe beam (orange line in Fig. 1a, b) is used to further promote the fluorophore from vibrationally excited state to electronically excited state and the following fluorescence emission is collected as the SREF signal (blue line in Fig. 1a, blue band in Fig. 1b). In our experiment, temporal-overlapped picosecond laser pulses are used to achieve efficient excitations (upper two rows in Fig. 1c). The fluorescence excitation spectrum as a function of the Raman shift (i.e., the SREF spectrum) maps out the Raman line shape of target Raman mode^20^ (red curve in Fig. 1d for the nitrile mode of rhodamine 800 (Rh800)). To simplify the excitation from a three-beam experiment to a two-beam experiment, the pump beam is also used as the probe beam in previous reports (the orange line in Fig. 1a, b).

Fundamentally SREF can be viewed as a fluorescence-detected vibrational spectroscopy. Our core idea is to apply STED to the fluorescence emission step of SREF for super-resolution imaging. Such coupling can be implemented conveniently with the reported SREF instrumentation. For efficient SREF excitation, the pump photon energy is set in the proper electronic pre-resonance range^21^. As shown in Fig. 1a, b, when this criterion is fulfilled, the pump wavelength is always at the tail of the fluorophore’s emission spectrum. Thus, a temporal-delayed new beam at the same pump wavelength can readily serve as a STED beam (after phase modulation by a phase plate) for the STED-SREF scheme (Fig. 1a–c). Hence, no new laser frequency component is required.

To test the feasibility of this direct STED-SREF strategy, a new STED beam is split from the pump beam, temporally delayed by ~6 ps, and finally spatially recombined with the pump (probe) and Stokes beams to achieve the excitation (Fig. 1c, Fig. S1). As expected, when ~4-mW 2-ps-pulsed STED beam with a Gaussian beam profile was applied, the SREF peak of the Rh800 nitrile mode was almost totally depleted (blue curve in Fig. 1d). However, about half of the overall fluorescence remained; further increasing the power of the STED beam resulted in even higher overall fluorescence (Fig. 1d). These observations can be explained by the origin of different fluorescence components in SREF spectrum^20,21^.

The function of the STED process is to drive the population of the electronically excited state equal to the thermal population (i.e., population obeying Boltzmann distribution under thermal equilibrium) of the intermediate vibrational excited state of the ground electronic state. In the absence of vibrational resonance, the pump (probe) beam excites anti-Stokes fluorescence background from the thermal population of intermediate vibrational excited state^20,21^, which clearly cannot be attenuated or depleted by the STED beam. In contrast, in the presence of vibrational resonance, the stimulated Raman process will create a “hot” vibrational population which gives rise to the pure SREF signal (the peak on a red curve in Fig. 1d). This additional population is created transiently by the pump and Stokes pulses beyond the thermal equilibrium (hence the term “hot”), and the associated pure SREF can certainly be depleted by the subsequent (delayed by ~6 ps) STED pulse. The two-photon fluorescence backgrounds are also created transiently beyond the anti-Stokes fluorescence component, and therefore also can be efficiently depleted. As a result, the remaining fluorescence emission in the direct STED-SREF scheme is mainly anti-Stokes fluorescence background. As the STED beam itself can also contribute to fluorescence background (both one-photon excitations and two-photon excitations), further increasing the STED beam power will only result in stronger overall fluorescence background (Fig. 1d). In regular STED fluorescence microscopy^4^, the population of the electronically excited state is much larger than the thermal population of the intermediate vibrational excited state. Therefore, the anti-Stokes fluorescence background can be omitted there. In contrast, in the STED-SREF case here, the “hot” population pumped to the electronically excited state by the SREF process is on a comparable level to the thermal population of the intermediate vibrational excited state.

After the spectroscopy investigation above, we then transformed the STED beam from a Gaussian mode to a standard donut shape by a vortex phase plate for imaging tests (Fig. S1). The imaging results of Rh800-stained *Escherichia* *coli* cells support the spectroscopic predictions. Because the anti-Stokes fluorescence background, which is the main component of the overall signal, cannot be attenuated (Fig. 1e, f), no obvious improvement on spatial resolution was achieved through the direct STED-SREF scheme (Fig. 1f, g, i). As a positive control, when only the STED beam and the Stokes beam were applied for imaging, the image constructed from two-photon fluorescence excited by the Stokes beam actually showed a significant resolution improvement (Fig. 1h, i), which also proves the effectiveness of the STED process and proper optical alignment. Together, these spectroscopy and imaging studies indicate that direct coupling SREF imaging with STED cannot achieve super resolution.

### Background-free SREF imaging through frequency modulation

We then sought to efficiently remove the anti-Stokes fluorescence background. The narrow line shape of a typical vibrational resonance (~10 cm^−1^) renders its sensitive response to the excitation frequency (i.e., the energy difference between pump and Stokes in the context of coherent Raman) (Fig. S2a), which has long been utilized to extract the vibrational signal from broadband backgrounds^24–33^. Technically, by temporally modulating the excitation frequency on- and off- the targeted vibrational resonance but still within the broad linewidth of the background, one can generate an intensity modulation on the vibrational signal (but not on the background) (Fig. S2a,b). Then, the background-free vibrational signal can be subsequently demodulated by the well-established lock-in detection technique. This strategy is called frequency modulation^24–33^ (FM).

To apply FM to SREF for background-free imaging, two main issues should be addressed regarding the excitation and detection, respectively. First, the anti-Stokes fluorescence component in the raw SREF spectrum follows a Boltzmann distribution (i.e., exponential trend) as the pump frequency changes^21^, resulting in a smooth but tilted background within several nanometers of pump wavelength tuning (Fig. 1d). In contrast, the Stokes photon energy is much further away from the electronic resonance (red line, Fig. 1b). So, compared to modulating the pump, modulating the Stokes frequency is preferred for background-free SREF application (Fig. S2a, b). We recently developed a simple and robust FM method for ps-pulsed laser-excited coherent Raman microscopy with superb noise property^24^. We will employ this strategy to fulfill the new scenario of FM-SREF (Fig. S2c). Briefly, self-phase modulation can induce self-balanced spectral splitting of picosecond Stokes beam propagating in standard single-mode silica fibers, resulting in two required wavelength-shifted Stokes beams. Second, SREF signal shall better be detected by single-photon counters rather than photomultiplier tubes because of the quantum yield consideration. However, because the typical output of single-photon counters is TTL-pulse sequence rather than analog currents, the conventional lock-in amplifier cannot be directly used for signal demodulation^34^. To solve this problem, we have developed a digital lock-in that is compatible with photon counting, based on commercially available devices (Fig. S3). With these engineering efforts, we successfully achieved FM-SREF microscopy (Fig. S2c).

**Fig. 2:**
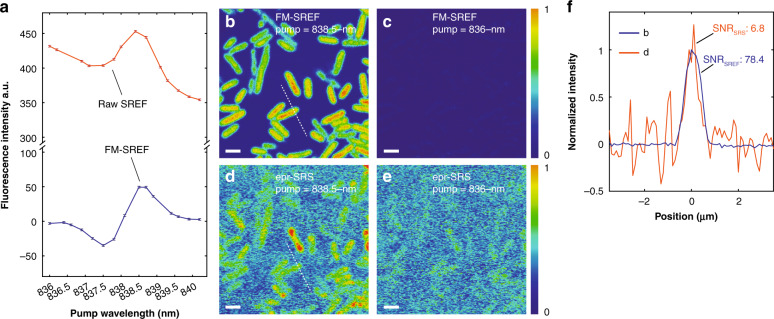
Background-free SREF spectroscopy and imaging achieved by frequency modulation (FM). **a** The raw SREF spectrum of Rh800 nitrile mode acquired by conventional SREF excitation (red curve) and corresponding background-free FM-SREF spectrum acquired by FM-SREF excitation (blue curve). 5-µM Rh800 DMSO solution was used. **b**, **c** The corresponding FM-SREF imaging of Rh800-stained *E. coli* cells with pump wavelengths set at 838.5 nm (nitrile mode on) and 83 nm (nitrile mode off). **d**, **e** The electronic pre-resonance stimulated Raman scattering (epr-SRS) imaging of the same field of view with pump wavelengths set at 838.5 nm (nitrile mode on) and 836 nm (nitrile mode off). All the configurations for the FM-SREF imaging and SRS imaging are the same. The conventional SREF excitation and epr-SRS excitation are achieved by block the blue-side Stokes beam (details in SI). For spectral measurements in (**a**), the pump (probe) power is set to 14 mW; The two Stokes beams are set to 6 mW each. For imaging in (**b**–**e**), the pump (probe) power was set to 5 mW; the two Stokes beams were set to 4 mW each. (**f**) shows the corresponding intensity distribution along the corresponding white dashed lines in (**b**) (blue curve) and (**d**) (red curve), respectively. Scale bar: 2 µm.

Figure 2 shows the performance of our FM-SREF microscopy. Comparing with the typical raw SREF spectrum that contains a strong tilted fluorescence background (red curve, Fig. 2a), the SREF spectrum acquired by FM-SREF represents the pure SREF signal, with all the fluorescence backgrounds totally subtracted (blue curve, Fig. 2a). The two peaks on the FM-SREF spectrum (blue curve, Fig. 2a) correspond to vibrational resonances with the blue-side Stokes beam (blue curve, Fig. S2d) and the red-side Stokes beam (red curve, Fig. S2d), respectively. The opposite signs of these two peaks are due to the π phase difference between the modulations of the two Stokes beams. Further FM-SREF imaging of *E.* *coli* cells stained by Rh800 also proves the robust background-free performance. Detectable SREF signals are observed only when the pump laser is tuning onto the vibrational resonance (Fig. 2b). In the off-resonance channel (Fig. 2c), the fluorescence backgrounds in conventional SREF imaging (Fig. 1e) are significantly suppressed. By coupling SRS excitation with fluorescence emission, SREF bypasses the strong laser shot noise encountered in the standard SRS detection, contributing to significant sensitivity improvement^20^. Indeed, when compared with the SRS imaging^35^ of the same field of view under the same excitation power and pixel dwell time (Fig. 2d–e), FM-SREF imaging achieved a signal-to-noise ratio (SNR) that is more than ten times better (Fig. 2b–f).

### Multicolor FM-SREF biological imaging with sharp vibrational contrast

**Fig. 3:**
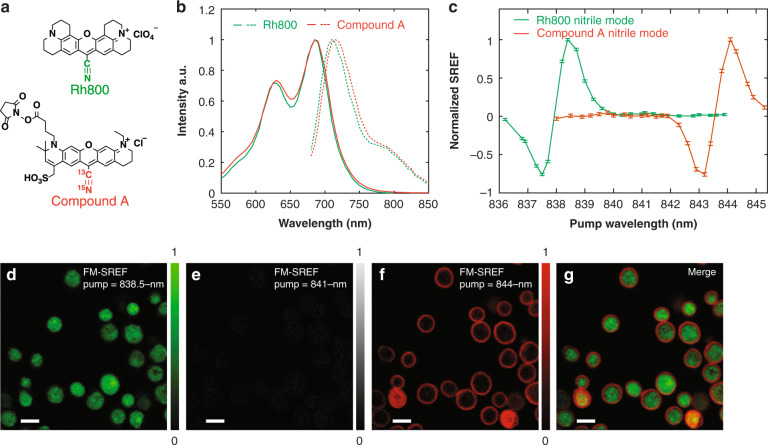
Multicolor FM-SREF biological imaging. **a** chemical structures of the two SREF dyes used in this work. **b** The absorption spectra (solid curves) and emission spectra (dash curves) of the two dyes in water. **c** The corresponding FM-SREF spectra of the nitrile modes of the two dyes. For (**b**, **c**), green curves for Rh800; red curves for compound A. **d**, **e**, **f** The corresponding FM-SREF image results with pump wavelengths set at 838.5 nm (the nitrile mode resonance of Rh800), 841 nm (the vibrational off-resonance for both Rh800 and compound A), and 844 nm (the nitrile mode resonance of compound A). **g** The merged results of (**d**) and (**f**). Scale bar: 5 µm

Harnessing the high contrast achieved above, we then aimed to demonstrate multicolor FM-SREF bio-imaging with sharp vibrational contrast. To obtain meaningful biological targeting, we synthesized a new SREF dye that is isotope edited (compound A in Fig. 3a, Details in SI). The absorption and emission of the new dye are nearly identical to that of Rh800 (Fig. 3b), and therefore can have the SREF signal of its nitrile mode (whose resonance is shifted by isotope editing) excited with our current laser system. Obviously, this dye cannot be used together with Rh800 for two-color imaging in conventional fluorescence microscopy. But with the intentional isotope editing of the nitrile bond, compound A can be readily distinguished from Rh800 by FM-SREF spectra (Fig. 3c).

To demonstrate bio-imaging, we stained the cytoplasm of the Saccharomyces *cerevisiae* cells by Rh800 (which is positively charged) through electrostatic interaction. By introducing a functional arm on compound A, we were able to stain the cell walls by a selective reaction of the N-hydroxysuccinimide (NHS) group in the arm with the amine groups on the cell walls. The additional introduction of the sulfonate group (–SO_3_H) on compound A was known to enhance the water solubility of the dye^36^, and it seems that the negative charge of the ionized sulfonate group also prevents the penetration of the dye into living *S. cerevisiae* cells. With our FM-SREF strategy, the corresponding pure SREF signals of the two dyes can be successfully imaged for two independent channels with their fluorescence backgrounds efficiently suppressed (Fig. 3d–g). When comparing with the conventional SREF imaging in which a hyperspectral imaging stack was acquired for probe identification^20^, here we only need to image the corresponding vibrational on-resonance wavelengths, which greatly improves the efficiency and reduces photobleaching induced by long time hyperspectral recording. It is important to note that these two channels are separated by FM-SREF with minimal cross-talk (Fig. 3d, f, g), while this will be extremely difficult (nearly impossible) by conventional fluorescence microscopy. This showcases the unique advantage of chemical specificity from vibrational imaging along multiplexed optical imaging^36^.

### Far-field super-resolution vibrational imaging achieved by STED-FM-SREF

**Fig. 4:**
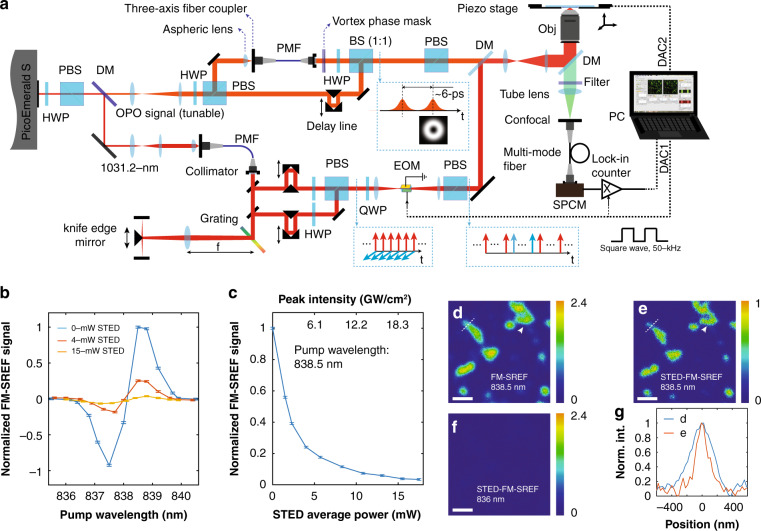
STED-FM-SREF microscopy. **a** The system diagram of STED-FM-SREF microscopy. **b** The FM-SREF spectra of the nitrile mode of Rh800 with the powers of STED beam set as 0 mW (blue curve), 4 mW (red curve), and 15 mW (orange curve), respectively. **c** The FM-SREF signal as a function of the power (and the peak intensity) of STED beam. The pump wavelength was set at 838.5 nm (i.e., the vibrational resonance of Rh800 nitrile mode). **d** the FM-SREF imaging of Rh800-stained PMMA nanostructures with pump wavelength set at 838.5 . **e**, **f** The corresponding STED-FM-SREF imaging of the same field of view of (**d**) with pump wavelengths set at 838.5 nm and 836 nm, respectively. **g** the intensity distributions along the dash lines in (**d**, **e**). For (**b**–**f**), the pump (probe) power was set to 5 mW; the two Stokes beams were set to 4 mW each. For (**e**, **f**), the STED beam was set to 6 mW. Scale bar: 1 μm

With successful background-free FM-SREF imaging, we revisited the approach of coupling STED for super-resolution vibrational imaging. Figure 4a shows the layout of our STED-FM-SREF microscope (detail in “Materials and methods”). Briefly, all the four beams (i.e., the pump beam which also serves as the probe beam, the two wavelength-shifted Stokes beams for FM, and the donut-shaped STED beam) can be prepared from the two laser outputs of the commercially available ps-pulsed OPO system (PicoEmerald S, APE). The 1031.2-nm near-IR picosecond pulse was coupled into a segment of polarization-maintaining single-mode silica fiber for self-phase modulation, which generated the two Stokes beams with ~1 nm wavelength difference. These two Stokes beams were then combined with orthogonal polarization and sent into an electro-optic modulator to achieve the FM of the Stokes beam. The tunable OPO signal pumped by the second harmonic generation of the 1031.2-nm laser pulse (inside the PicoEmerald S laser box) was split into two beams. One of the beams was directly used as the pump beam (also the probe beam). The spatial profile of the other beam was first filtered by a piece of single-mode fiber and then transformed to the standard donut shape by a vortex phase plate. This donut-shaped STED beam is then temporally delayed by ~6 ps before recombined with the pump (probe) beam. Finally, the pump (probe) beam, two Stokes beams, and the donut-shaped STED beam were spatially combined (with the first three pulse trains temporally overlapped) and sent into an inverted microscope for STED-FM-SREF. Sample scanning was achieved by a piezo stage. The emitted fluorescence was confocally detected in the backward direction. A single-photon counting module (SPCM) coupled with a home-built digital lock-in was used to demodulate the pure SREF signal via FM-SREF.

With this new setup, we finally achieved the desirable behavior of fluorescence depletion by the donut-shaped beam^4^ (Fig. 4b, c). The highly nonlinear relationship between the remaining fluorescence intensity and the power of the donut-shaped STED beam is a prominent signature for the feasibility of resolution improvement (Fig. 4c). Further STED-FM-SREF imaging of the Rh800-stained PMMA nanostructures (details in “Materials and methods”) confirmed this prediction. As shown in Fig. 4d, e, when excited at the vibrational resonance of the nitrile mode of Rh800, the structure with the smallest full width at half maximum (FWHM) is ~400 nm under FM-SREF, whereas the same structure shows a ~180 nm FMHM under STED-FM-SREF with a modest STED power (6 mW) (Fig. 4g), indicating a more than two times improvement of the resolution. With such a resolution improvement, more details of some nanostructures can be revealed (such as the structure pointed by the white arrow in Fig. 4d, e). As a control, STED-FM-SREF imaging of the same structure with pump wavelength set at the off vibrational resonance of the nitrile mode shows no net signal (Fig. 4f), which again confirmed the background-free property of our FM strategy coupled with the additional STED excitation.

**Fig. 5:**
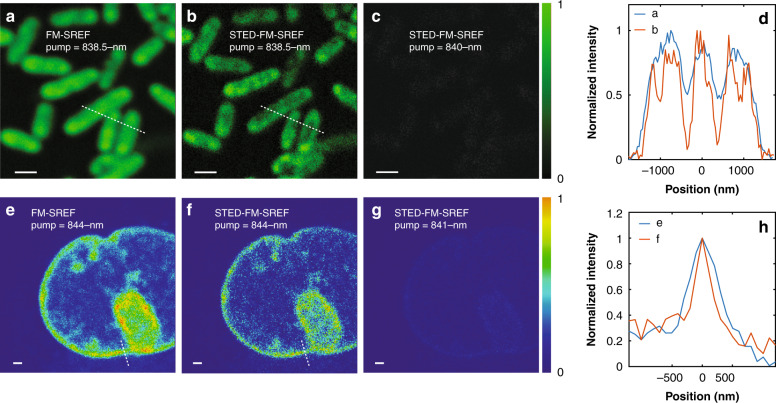
Vibrational imaging of biological systems by STED-FM-SREF. **a**, **b** The images of the same Rh800-stained *E. coli* cells by FM-SREF microscopy and STED-FM-SREF microscopy with pump wavelength set at the vibrational resonance of the nitrile mode of Rh800 (i.e., 838.5 nm), respectively. **c** The imaging of the same field of view of (**a**, **b**) by STED-FM-SREF microscopy with pump wavelength set at the off resonance of the nitrile mode of Rh800 (i.e., 836 nm). **d** The intensity distributions along the dash lines in (**a**, **b**). **e**, **f** Corresponding images of the compound A-stained Cos7-cell nucleus by FM-SREF microscopy and STED-FM-SREF microscopy with pump wavelength set at the vibrational resonance of the nitrile mode of compound A (i.e., 844 m), respectively. **g** Imaging of the same field of view of (**e**, **f**) by STED-FM-SREF microscopy with pump wavelength set at the off resonance of the nitrile mode of compound A (i.e., 841 nm). **h** The intensity distributions along the dash lines in (**e**, **f**). For all measurements, the pump (probe) power was set to 5 mW; the two Stokes beams were set to 4 mW each; the STED beam was set to 6 mW. Images are normalized to the same gray-level scale for the convenience of comparation. Scale bar: 1 μm

To further demonstrate the feasibility of our method for diverse biological samples, we achieved super-resolution vibrational imaging by STED-FM-SREF in both bacterial and mammalian cells. Figure 5 shows the typical results achieved on structures labeled by Rh800 and our newly synthesized compound A. First, we confirmed that no details inside the Rh800-stained *E. coli* cells (with width ~500-nm) can be resolved (Fig. 5a) under the diffraction-limited resolution of FM-SREF imaging (~400 nm for our system, as shown in Fig. 4d, g). After breaking the diffraction limit by STED, STED-FM-SREF imaging reveals an obvious chamber with a lower SREF signal inside *E.coli* cell interiors (Fig. 5b, d), indicating an inhomogeneous nonspecific-binding pattern of Rh800 molecules. This is consistent with the previously reported pattern of Pyridine 4 stained *E. coli* cell captured under standard STED microscopy^2^, as well as our own image captured by two-photon STED (Fig. 1h). Also, tuning the pump wavelength only by 1.5-nm off the vibrational resonance of the nitrile mode totally diminishes the signal (Fig. 5c), which again shows the chemical specificity of our super-resolution vibrational imaging. With the functional moiety on compound A, we employed click chemistry to specifically label the cellular DNA molecules inside the nucleus of *Cos7* mammalian cells (details in ”Materials and methods”). Again, the STED-FM-SREF image shows richer textures and sharper edges than that of the FM-SREF image and exhibits spectrally sharp vibrational contrast at the same time (Fig. 5e–h). These results proved STED-FM-SREF as a biocompatible super-resolution vibrational imaging technique.

## Discussion

Insufficient sensitivity has long been the bottleneck of vibrational imaging, which largely accounts for the difficulty of pushing vibrational imaging beyond the diffraction limit. The recently developed SREF drastically improves the detection sensitivity of vibrational modes through the combined effects of stimulated Raman excitation and fluorescence detection. Considering the STED technique has been well established in breaking the diffraction limit for fluorescence microscopy, coupling STED with SREF might seem a straightforward strategy. Yet we showed that direct application of STED to SREF imaging did not result in resolution improvement. This is because the co-excited anti-Stokes fluorescence background originated from thermal equilibrium, cannot be depleted (Fig. 1). By applying FM on the Stokes beam and demodulating the detected fluorescence, we achieved background-free SREF microscopy (Fig. 2) and further demonstrated multicolor FM-SREF imaging in cells with nm-level spectra resolution with newly synthesized SREF dyes (Fig. 3). Finally, we achieved far-field super-resolution vibrational imaging (Fig. 4) through an integrated STED-FM-SREF scheme. Compared to most of the reported super-resolution Raman methods, better resolution enhancement was achieved here with a lower laser power in the demonstrated biological imaging (Figs. 4 and 5).

SREF is unique among all vibrational imaging techniques in that it employs fluorescent probes, which can be regarded as a “double-edged sword” to some extent. On one hand, the reliance on fluorescent probes might be perceived as less general than the conventional label-free paradigm in vibrational imaging, as not all molecules are fluorescent by nature. On the other hand, it is the fluorescence detection that renders the superb (down to single-molecule level) sensitivity of SREF spectroscopy^20^. Moreover, fluorescent probes can be introduced to label specific molecular targets or structures or to serve as spectroscopy sensors of the local environment, as demonstrated in recent SREF applications of biophotonics, biophysics, and materials science^22,23^ as well as the SREF dye staining presented here (Figs. 3 and 5). Local environmental sensing (such as electric field and hydrogen bonding) and super-multiplexed vibrational imaging are two prominent classes of applications in which introducing specific probes can bring clear advantages. Hence, as any techniques have their own benefits and drawbacks, SREF based super-resolution imaging is no exception.

As well established in STED microscopy, the ultimate resolution limit of STED-FM-SREF imaging is theoretically determined by the obtainable SNR of FM-SREF signal, which is mainly set by the ratio between the pure FM-SREF signal and the shot noise of the overall fluorescence background. The millisecond-long pixel dwell time, currently required by the slow speed of our stage scanner and data-acquisition electronics, causes detectable photobleaching and thus prevents us from getting better SNR. Future adoption of faster laser scanning and acquisition electronics could lessen the photobleaching problem. More importantly, limited by the current laser source constructed from a commercial system, only near-IR dyes can be efficiently excited by FM-SREF. The low quantum yields of near-IR dyes (~4% for Rh800 in water^37^, and the quantum yield of compound A is comparable to Rh800) result in relatively poor SNR and thus limit the resolution enhancement in our current demonstrations. Future adoption of more flexible laser sources in the visible or even UV region is expected to open up a wider range of target molecules with brighter emission and hence further improve the resolution. With future SREF probe palette design and instrumental optimization, we anticipate applications of STED-FM-SREF microscopy on a variety of systems.

## Materials and methods

### System construction

The details of the frequency-modulation method we adopt here can be found in our previous publication^24^. The main difference comes from the signal detection part. For FM-SREF detection, a non-resonance electro-optic modulator (EO-AM-NR-C2, Thorlabs) is used, and it is driven by a 50-kHz square wave amplified by a high-voltage amplifier (HVA200, Thorlabs) to achieve more than 90% modulation depth. The square wave is generated by our home-built lock-in photon counter for the convenience of phase control and signal synchronization. For filter set configuration, a 2-mm-thick shortpass dichroic mirror (T785spxxr-UF2, Chroma) was used for flatness consideration, all the other optical filters used for nitrile-band SREF signal detection are the same as those used in our previous publication^20^. The same objective (UPLSAPO, 1.2NA, Olympus) and detector (SPCM-NIR-14-FC, Excelitas) were used for all measurements and imaging. And all imaging scanning and data acquisition (including the lock-in photon counter) are driven by a Multifunction I/O card (USB-6259, NI) controlled with a LabVIEW-based home-built software. The detailed construction of the three systems used in this research can be found in Fig. S1, Fig. S2c, and Fig. 4a. The lock-in photon counter can be coded by following the time sequence diagram shown in Fig. S3.

### Sample preparation

Four different types of samples were used in this research. The corresponding protocols are listed below.

#### The preparation of Rh800-stained *E. coli* cells

The cells were first cultured with 3-μM Rh800 water solution for ~10 min, and rinsed by distilled water, then fixed on a polylysine-coated coverslip with 4% PFA solution; at last, the sample was sealed by an imaging spacer (GBL654008, Sigma) with the chamber filled by distilled water.

#### The preparation of Rh800-stained PMMA nanostructures

First, 100-μL 1% PMMA (the powder in Technovit 9100, EMS) toluene (650579, sigma) solution and 2-μL hexadecane (52209, sigma) were mixed; then 20-μL of the mixture was spin-coated on a standard coverslip with 5000-rpm speed to generate the nanostructure (by phase separation during the fast evaporation of toluene); at last, 3-μL 10-mM Rh800 DMSO solution was spin-coated on top of the previous coating layer with the same speed to achieve the staining. The sample was sealed by an imaging spacer (GBL654006, sigma) with the chamber filled with hexadecane.

#### The preparation of multicolor *S. cerevisiae* (the YPH499 strain) sample

The cells were cultured in YPAD medium at 30 °C overnight, rinsed, and resuspended in the sodium bicarbonate PBS buffer (pH 8.3). To stain the cell wall, we added 3-μL compound A DMSO solution (2 mM) in 20-μL cell solution and incubate the mixture at 42 °C for 15 min. After this, the cells were rinsed by PBS and resuspended in 20-μL poly-d-Lysine solution (0.1 mg/mL, A3890401, Fisher Scientific). We then spin-coated the cell suspension onto a polylysine-coated coverslip (700 rpm, 10 min) for uniform distribution and better adhesion, and fixed the cells on the coverslip with 4% PFA solution for 15 min at 37 °C. After rinsed twice by PBS, the sample was incubated in 10-μM Rh800 PBS solution for 5 min at 37 °C to stain the cell cytoplasm. At last, the sample was rinsed with distilled water and sealed by an imaging spacer (GBL654008, Sigma) with the chamber filled with distilled water for the following imaging.

#### The preparation of compound A-stained Cos7-cell DNA sample

*Cos7* cells were first seeded on coverslips, and cultured with Dulbecco’s Modified Eagle’s Medium (DMEM, 11965) supplemented with 10% FBS and 1% penicillin–streptomycin to 30% confluency. The culture medium was then changed into DMEM (FBS-free) for 24 h to synchronize the cell cycles. The cellular DNA was then labeled by culturing the cells in a full DMEM culture medium supplemented with 100-μM EdU for another 15 h. After rinsed twice by PBS, the cells were pre-extracted and fixed with extraction buffer [i.e., 80-mM PIPES (pH 6.9), 7-mM MgCl_2_, 1-mM EGTA, 0.3% Triton-X100, 150-mM NaCl, 5-mM glucose, 0.25% glutaraldehyde] at 37 °C for 90 s, and further fixed by 4% PFA at 37 °C for 10 min. The fixed cells were then rinsed three times by PBS and permeabilized with 0.3% Triton-X100 for 30 min. After all these pre-preparation processes, the cells were rinsed three times by PBS for Compound A staining via click chemistry. One microliter 10-mM Azido-PEG_4_-NHS Ester (Fisher Scientific, A238825MG) DMSO solution and 1-μL 10-mM PAMAM dendrimer (Sigma, 412368) DMSO solution were mixed in 8-μL DMSO and incubated at 45 °C for 30 min, and then mixed with 30-μL PBS and 10-μL 2-mM compound A DMSO solution. The mixed solution was incubated at 45 °C for another 0.5 h to obtain the compound A DNA staining solution. Fifteen microliters obtained solution was further mixed with Click-iT cell reaction reagents (Invitrogen, C10259) to achieve a total volume of 375 μL. This final solution was then used to label the EdU incorporated in the Cos7 cells by 2 h incubation at room temperature. The sample was then washed and blocked for 30 min with 5% BSA solution containing 0.1% Triton. Finally, the sample was rinsed with distilled water and sealed by an imaging spacer (GBL654008, Sigma) with the chamber filled with oxygen scavenger solution. The oxygen scavenger solution was prepared by mixing 6-μL 1.5-M Tris-HCl (pH 8.8), 20-μL 50% glucose solution, 73-μL distilled water, and 2-μL mixed enzyme solution containing 2% (w/v) glucose oxidase enzyme (Sigma, G2133) and 1.5% (w/v) catalase (Sigma, C40).

### Imaging configurations

For all FM-SREF imaging and SRS imaging of *E. coli* cells, the pixel size was set to 100 nm, the pixel dwell time was set to 1 ms and a two-frame of average is applied. For the two-color FM-SREF imaging, the pixel size was set to 180 nm, the pixel dwell time was set to 1 ms and a two-frame average was applied. For STED-FM-SREF imaging of Rh800-stained PMMA nanostructures, the pixel size was set to 40 nm, the pixel dwell time was set to 1 ms and a two-frame average was applied. For STED-FM-SREF imaging of Rh800-stained *E. coli* cells, the pixel size was set to 40 nm, the pixel dwell time was set to 1.2 ms and a four-frame average was applied. For STED-FM-SREF imaging of compound A-stained *Cos7* cells, the pixel size was set to 100 nm, the pixel dwell time was set to 1 ms and a three-frame average was applied. All the laser power configurations were mentioned in the corresponding figure captions.

## Supplementary information

Supplementary Information

## Data Availability

The data and code used in this research are available from the corresponding author upon reasonable request.
