# Supplementary material for: Super-resolution vibrational microscopy by stimulated Raman excited fluorescence
Source: Light Sci Appl. 2021 Apr 20;10:87. doi: 10.1038/s41377-021-00518-5 (PMC8058038; doi:10.1038/s41377-021-00518-5)
Supplement: Supplementary file 1 — Supplementary Information [file 41377_2021_518_MOESM1_ESM.docx]

# Supplementary Information for

# Super-resolution vibrational microscopy by stimulated Raman excited fluorescence

Hanqing Xiong^†^, Naixin Qian^†^, Yupeng Miao, Zhilun Zhao, Chen Chen, Wei Min*

Department of Chemistry, Columbia University, New York, NY 10027, USA

* Corresponding author. E-mail: [wm2256@columbia.edu](mailto:wm2256@columbia.edu)

^†^ These authors contribute equally to this work

**This file contain the following contents:**

Figure S1-S3

Synthesis of a new SREF dye


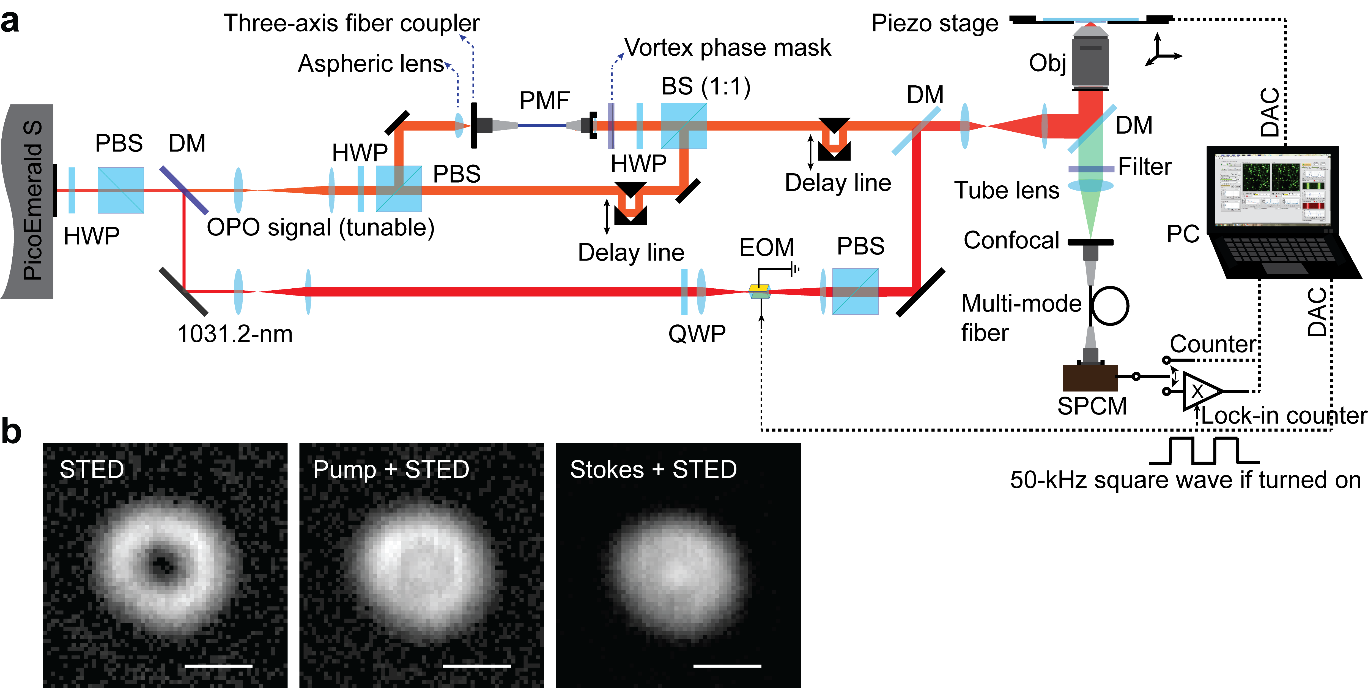


Fig. S1 Direct coupleing between stimulated depletion emission (STED) and stimulated Raman excited fluorescence (SREF) microscopy. (a) the diagram for the STED-SREF system. As indicated by previous pulse-STED related studies^1^, the time delay between the pluses used for SREF excitation and the STED pulse should be set large enough to avoid temporal overlap, and small enough to avoid obvious spontaneous emission before the STED pulse arrives (i.e., much smaller than the fluorescence lifetime). 6-ps time delay is used here for our 2-ps pulsed laser source. HWP for half-wave plate; PBS for polarization beam splitter; DM for dichroic mirror; OPO for optical parametric oscillator; PMF for polarization-maintaining single-mode fiber; BS for beam splitter, here the transmission and reflection ratio is 1:1; QWP for quarter-wave plate; EOM for electro-optic modulator; Obj for objective lens; SPCM for single photon counter module; PC for personal computer; DAC for digital-to-analog converter. (b) shows the beam profiles of the donut-shaped STED beam, the overlap of pump beam with STED beam, and the overlap of STED beam with Stokes beam mapped out by the luminescence of Ag nanoparticles, respectively. Scale bar: 500 nm. Note that the vortex phase mask is removed for all the spectroscopy experiments in Fig. 1; and except for STED-two-photon fluorescence imaging (Fig. 1h), the EOM is turned off, and the SPCM is connected to the counter rather than lock-in counter.


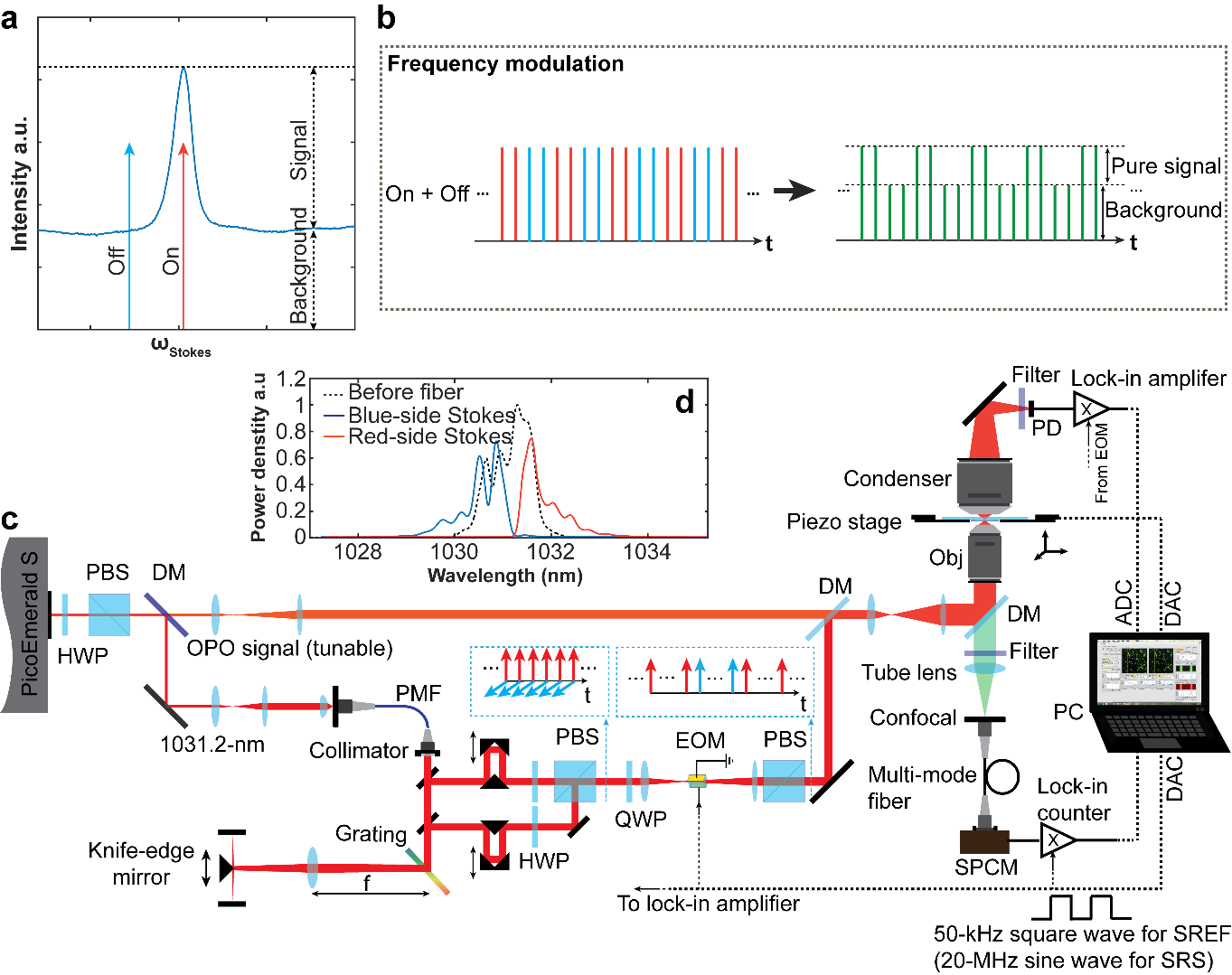


Fig. S2 Frequency-modulated (FM) stimulated Raman excited fluorescence (SREF) microscopy. (a) and (b) show the principle of background-free coherent Raman detection by frequency modulation of Stokes beam. Note that the pure signal is extracted by lock-in detection. (c) shows the system diagram of our FM-SREF microscope. HWP for half-wave plate; PBS for polarization beam splitter; DM for dichroic mirror; OPO for optical parametric oscillator; QWP for quarter-wave plate; EOM for electro-optic modulator; PMF for polarization-maintaining single-mode fiber; Obj for objective lens; PD for photodiode; SPCM for single photon counter module; PC for personal computer; DAC for digital-to-analog converter; ADC for analog-to-digital converter. Note that when this system is used for stimulated Ramana scattering imaging, the modulation of the EOM changed from 50-kHz square wave to 20-MHz sine wave to achieve the sensitivity approaching the shot-noise limit. (d) The spectra of the two Stokes beams used for FM. Blue curve for the blue-side Stokes beam; red curve for the red-side Stokes beam; Dashed black curve for the laser IR output before coupling to the fiber.


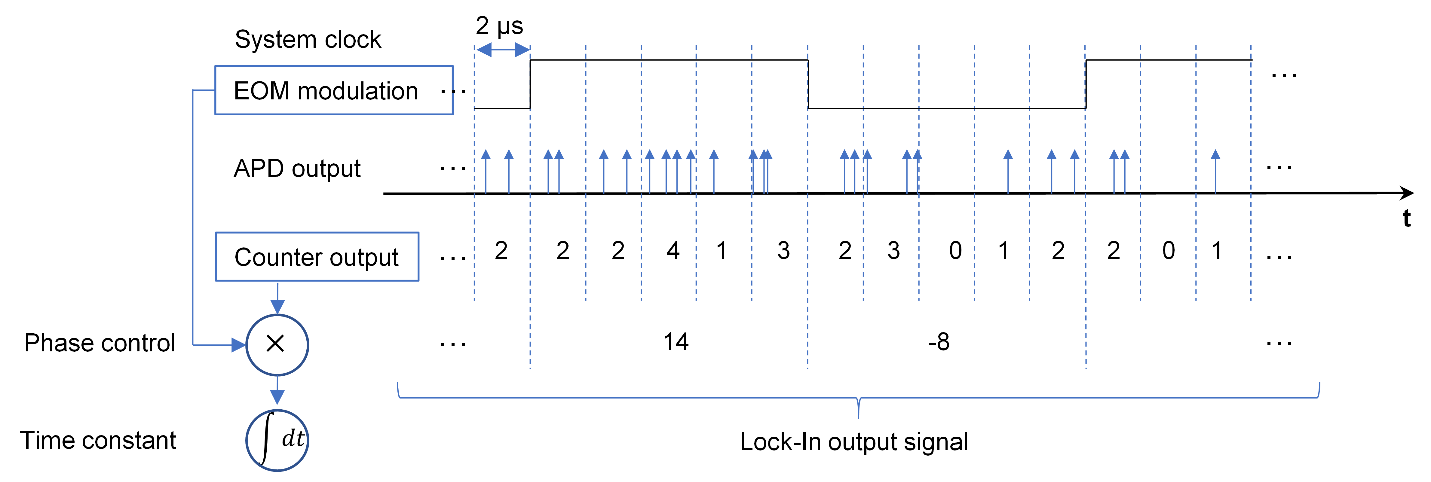


Fig. S3 The time sequence diagram of the home-built Lock-in photon counter realized on a NI card (USB 6259, NI). Note that the electro-optic modulator (EOM) is driven by a square wave with a period even times of the system clock period; the time constant must be integer times of the EOM modulation period. The whole system (including the imaging scanning) is synchronized to the system clock.

## Synthesis of a new SREF dye


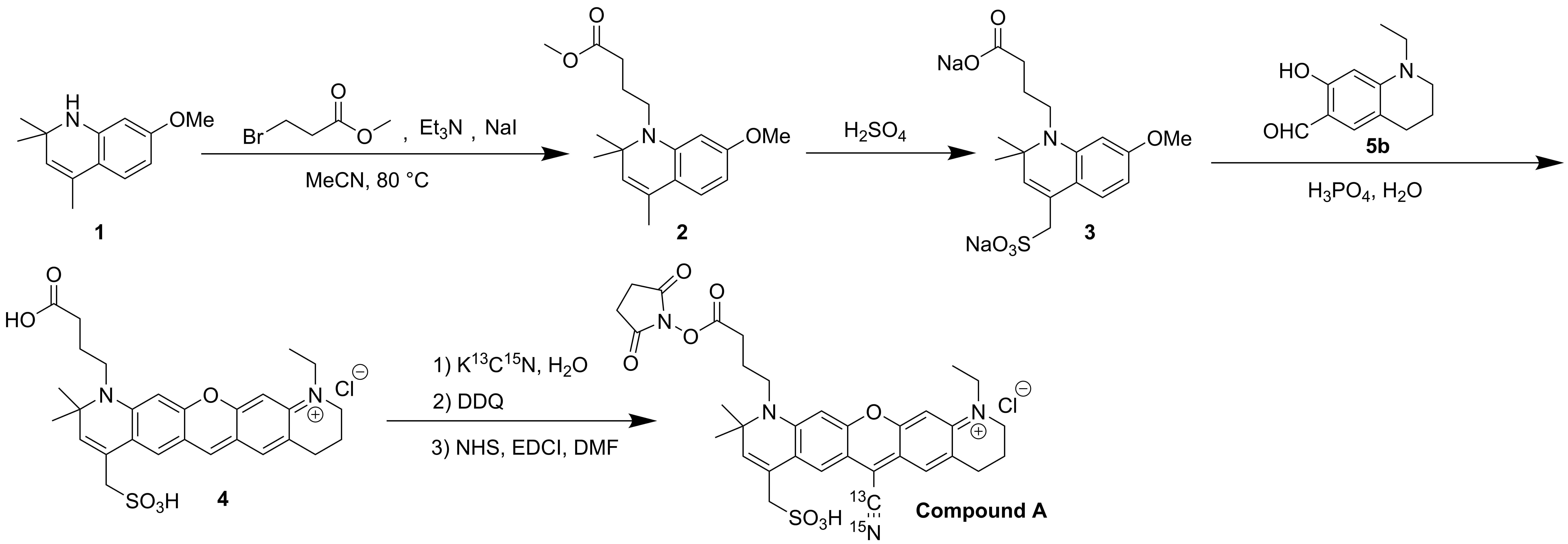

**7-methoxy-2,2,4-trimethyl-1,2-dihydroquinoline (1).** 1 is synthesized according to reported protocol.^2^

**methyl 4-(7-methoxy-2,2,4-trimethylquinolin-1(2H)-yl)butanoate (2).** Compound 1 (200 mg, 0.98 mmol) was dissolved in MeCN (5 mL) before NaI (21 mg, 0.14 mmol), Methyl 4-bromobutyrate (350 μL, 2.87 mmol) and triethylamine (400 μL, 5.5 mmol) were added. The reaction was heated to 80 °C and refluxed overnight. Water was added after cooling to room temperature. The mixture was extracted with methylene chloride for three times. The combined organic layer was washed with 2 N HCl, and dried over Na_2_SO_4_ before evaporation. The obtained residue was then purified by silica gel flash chromatography (EtOAc/ Hexane = 5%-15%) to obtain compound 2 as light-yellow oil (80 mg, 27%).

^1^H NMR (500 MHz, CDCl_3_) δ 6.97 (d, *J* = 8.0 Hz, 1H), 6.23 – 6.10 (m, 2H), 5.11 (s, 1H), 3.80 (s, 3H), 3.69 (s, 3H), 3.26 – 3.19 (m, 2H), 2.39 (t, *J* = 7.0 Hz, 2H), 1.99 – 1.87 (m, 5H), 1.27 (s, 6H).

^13^C NMR (126 MHz, CDCl_3_) δ 173.8, 160.7, 145.3, 127.6, 127.3, 124.6, 117.1, 99.5, 98.1, 57.0, 55.3, 51.8, 43.5, 31.5, 28.4, 23.5, 18.9, 14.3.

HRMS (ESI+) m/z Calcd. for C_18_H_26_NO_3_^+^ [M+H]^+^ : 304.1913. Found: 304.1926

**Sodium 4-(7-methoxy-2,2-dimethyl-4-(sulfonatomethyl)quinolin-1(2H)-yl)butanoate (3).** The compound 2 (53.1 mg, 0.175 mmol) was added to pre-cooled sulfuric acid (2 mL) and carefully sonicated to fully dissolve the reactant. The reaction was stirred at 0°C and left to gradually returned to room temperature overnight. The reaction was again cooled to 0 °C before water (2 mL) was added to dilute the solution. Saturated NaOH solution was carefully added to the red solution while stirring at 0 °C until the color changed into light yellow. The pH of the solution became greater than 10. Water was evaporated under vacuum and the residue was resuspended in ethanol. The obtained mixture was refluxed for 1h before filtrated through a silica pad with ethanol as eluent. Ethanol was further evaporated and the obtained residue was purified with reverse-phase chromatography (MeCN/ H_2_O= 30% - 100%) to obtain compound 3 as light-yellow solid (62 mg, 95%).

^1^H NMR (500 MHz, MeOD) δ 7.24 (d, *J* = 8.4 Hz, 1H), 6.19 (d, *J* = 2.4 Hz, 1H), 6.15 (dd, *J* = 8.5, 2.4 Hz, 1H), 5.46 (s, 1H), 3.84 (s, 2H), 3.75 (s, 3H), 3.30 – 3.23 (m, 2H), 2.38 (t, *J* = 6.9 Hz, 2H), 1.85 (p, *J* = 7.0 Hz, 2H), 1.32 (s, 6H).

^13^C NMR (126 MHz, MeOD) δ 177.6, 162.0, 146.7, 133.0, 126.8, 125.7, 116.7, 101.5, 98.9, 58.0, 55.6, 54.8, 44.5, 32.4, 28.0, 24.8.

HRMS (ESI+) m/z Calcd. for C_17_H_21_NO_6_SNa_3_^+^ [M+Na]^+^ : 436.0783. Found: 436.0775

**Pyronin intermediate (4).** The compound 3 (62 mg, 0.168 mmol) and compound 5b (34 mg, 0.168 mmol) was dissolved in 2 mL 85% phosphoric acid. The reaction was heated up to 100 °C and stirred overnight. Th reaction was then cooled to room temperature before poured into ice. The resulting aqueous solution was extracted with DCM and methanol in combination to increase the solubility. The organic phase was combined and dried over Na_2_SO4. The solvent was evaporated under vacuum. The obtained residue was purified with flash chromatography (MeOH/ DCM =10% - 50%) to obtain compound 4 as purple solid (12 mg, 13%).

^1^H NMR (400 MHz, MeOD) δ 8.09 (s, 1H), 7.70 (s, 1H), 6.95 (s, 1H), 6.74 (s, 1H), 5.88 (s, 1H), 5.49 (s, 1H), 4.02 (s, 2H), 3.63 – 3.53 (m, 6H), 2.90 – 2.79 (m, 4H), 2.49 – 2.43 (m, 2H), 2.01 – 1.96 (m, 5H), 1.56 (s, 6H).

^13^C NMR (101 MHz, MeOD) δ 169.7, 158.7, 158.0, 156.8, 154.8, 154.2, 138.6, 127.7, 126.8, 124.7, 124.5, 122.7, 115.2, 114.9, 96.9, 95.7, 61.4, 55.1, 50.4, 47.8, 46.4, 33.4, 30.7, 29.4, 28.6, 24.4, 22.2.

HRMS (ESI+) m/z Calcd. for C_28_H_33_N_2_O_6_S^+^ [M+H]^+^ : 525.2059. Found: 525.2048

**Compound A.** The compound 4 (8.4 mg, 0.016 mmol) was dissolved in DMF (2 mL) and KCN solution in H_2_O (0.1 M, 680 μL, 0.068 mmol) was added dropwise to the solution under stirring. The reaction was stirred at room temperature for 20 min until the pink color turned into light blue. 2,3-Dichloro-5,6-dicyano-1,4-benzoquinone (18.2 mg, 0.08 mmol) was added to oxidize the intermediate and the reaction was kept stirring for another 2 hours at room temperature. DMF was then evaporated and the obtained residue was dissolved in water for reverse-phase chromatography (MeCN/ H_2_O= 0% - 100%). The obtained blue solid, N-hydroxylsuccinimide (2.1 mg, 0.018 mmol), and 1-ethyl-3-(3-dimethylaminopropyl)carbodiimide (4.7 mg, 0.03mmol) was dissolved in DMF (1 mL) and stirred at room temperature overnight. DMF was evaporated under vacuum and the residue was purified with reverse-phase chromatography (MeCN/ H_2_O= 0% - 100%) to obtain Compound A as blue solid (1 mg, 11%).

^1^H NMR (500 MHz, DMSO) δ 7.90 (s, 1H), 7.58 (s, 1H), 7.22 (s, 1H), 7.11 (s, 1H), 5.94 (s, 1H), 3.80 – 3.63 (m, 6H), 3.17 (s, 2H), 3.01 (t, *J* = 7.3 Hz, 2H), 2.92 (t, *J* = 6.1 Hz, 2H), 2.84 (s, 4H), 2.02 – 1.90 (m, 4H), 1.52 (s, 6H), 1.27 – 1.22 (m, 3H).

HRMS (ESI+) m/z Calcd. for C_32_^13^CH_35_N_3_^15^NO_8_S^+^ [M+H]^+^ : 649.2180. Found: 649.2181

**1-ethyl-1,2,3,4-tetrahydroquinolin-7-ol (5a). 5a** was synthesized following previously published procedures.^3^

**ethyl-7-hydroxy-1,2,3,4-tetrahydroquinoline-6-carbaldehyde (5b).** To an Ar flushed flask was added 4 mL anhydrous N, N-dimethylformamide (DMF). After cooled to 0 °C, phosphoryl chloride (POCl_3_, 0.33 mL, 3.6 mmol, 1.2 eq) was added to DMF and kept stirring for 30 min followed by the addition of **5a** (531 mg, 3.0 mmol) in 4 mL dry DMF. The temperature was raised to 90 °C and kept for 5 h while stirring. After cooled to room temperature, the reaction was quenched upon addition of 30 mL iced water and the pH was adjusted to 8 with solid NaHCO_3_. The aqueous phase was extracted with ether (50 mL ×3) and the combined organic layers were dried over Na_2_SO_4_. The solvents were removed in vacuo to afford **5b** as brown solid, which has sufficient purity to be used in next step (461 mg, 75%).

^1^H NMR (500 MHz, CDCl_3_) δ 11.64 (s, 1H), 9.43 (s, 0H), 7.00 – 6.94 (m, 1H), 6.04 (s, 1H), 3.43 – 3.34 (m, 4H), 2.69 (td, *J* = 6.1, 1.0 Hz, 2H), 1.95 (dq, *J* = 7.4, 5.9 Hz, 2H), 1.21 (t, *J* = 7.1 Hz, 3H). ^13^C NMR (126 MHz, CDCl_3_) δ 191.5, 163.4, 151.9, 133.0, 114.8, 110.9, 95.5, 48.7, 46.0, 27.2, 21.8, 11.1. HRMS (ASAP+) m/z Calcd. for C_12_H_16_NO_2_ [M+H]^+^: 206.1181. Found: 206.1180

## Refferences

1. Galiani, S.; Harke, B.; Vicidomini, G.; Lignani, G.; Benfenati, F.; Diaspro, A.; Bianchini, P., Strategies to maximize the performance of a STED microscope. *Opt. Express* **2012,** *20* (7), 7362-7374.

2. Pauff, S. M.; Miller, S. C., Synthesis of Near-IR Fluorescent Oxazine Dyes with Esterase-Labile Sulfonate Esters. *Organic letters* **2011,** *13* (23), 6196-6199.

3. Anzalone, A. V.; Wang, T. Y.; Chen, Z.; Cornish, V. W., A common diaryl ether intermediate for the gram-scale synthesis of oxazine and xanthene fluorophores. *Angew Chem Int Ed Engl* **2013,** *52* (2), 650-4.
